# Efficacy and safety of lorundrostat in uncontrolled hypertension: a meta-analysis

**DOI:** 10.1007/s00210-025-04726-3

**Published:** 2025-11-18

**Authors:** Alaa Abdrabou Abouelmagd, Shrouk Ramadan, Mohamed Saad Sayed, Omar Abdulrahman Saad, Mazen Negmeldin Yassin, Alaa Mohammed Mostafa, Kerollos Abdelsayed

**Affiliations:** 1 Faculty of Medicine, South Valley University, Qena, Egypt; 2 Faculty of Medicine, Ain Shams University, Cairo, Egypt; 3 Faculty of Medicine, Beni-Suef University, Beni Suef, Egypt; 4 Faculty of Medicine, Al-Azhar University, Cairo, Egypt; 5 Faculty of Medicine, Cairo University, Cairo, Egypt; 6 Heart Rhythm Science Center Minneapolis Heart Institute Foundation, Minneapolis, MN USA

**Keywords:** Lorundrostat, Uncontrolled hypertension, Aldosterone synthase inhibitor, Meta-analysis, Hyperkalemia, Resistant hypertension

## Abstract

Lorundrostat, a novel selective aldosterone synthase inhibitor, has emerged as a potential treatment for uncontrolled hypertension. However, a comprehensive summary of its efficacy and safety profile is currently lacking. This meta-analysis aims to synthesize evidence from published randomized controlled trials (RCTs) to evaluate the clinical benefits and risks of lorundrostat in this patient population. Following the PRISMA guidelines, a systematic search was conducted on PubMed, Scopus, and Web of Science for RCTs comparing lorundrostat with placebo. The primary outcomes were changes from baseline in systolic and diastolic blood pressure. Data were pooled using a random-effects model to calculate mean differences (MD) for continuous outcomes and risk ratios (RR) for dichotomous outcomes, with corresponding 95% confidence intervals (CIs). Three RCTs comprising 1568 patients were included. Compared to placebo, lorundrostat significantly reduced both 24-h systolic blood pressure (MD: − 6.65 mmHg; 95% CI: [− 9.95 to − 3.35]) and office systolic blood pressure (MD: − 7.94 mmHg; 95% CI: [− 9.72 to − 6.15]). While the risk of serious adverse events was comparable, lorundrostat was associated with a higher incidence of hyperkalemia (RR: 5.47; 95% CI: [1.55 to 19.36]) and hyponatremia (RR: 1.95; 95% CI: [1.13 to 3.36]). Notably, lorundrostat significantly reduced the risk of adrenal insufficiency (RR: 0.15; 95% CI: [0.03 to 0.91]). Lorundrostat may be an effective agent for lowering blood pressure in patients with uncontrolled hypertension. Its safety profile is acceptable but requires careful monitoring of serum electrolytes and renal function. The reduced risk of adrenal insufficiency highlights its selectivity and potential as a valuable therapeutic option. Further large-scale, long-term studies are needed to confirm its sustained benefits and safety.

## Introduction

Standing as a significant risk factor for cardiovascular and cerebrovascular disease, kidney failure, and even early death, hypertension is a major public health issue globally, with approximately 1.13 billion individuals affected (Hsu et al. 2005; Kannel 2009; NCD Risk Factor Collaboration (NCD-RisC) 2017). Uncontrolled hypertension remains a challenge for many patients despite advancements in pharmacotherapy (Ritchie et al. 2013). The existing management protocols place a growing focus not only on effective control of hypertension, but also early recognition and purposeful treatment of the pathophysiological processes (Unger et al. 2020; Carey et al. 2021; Zuin et al. 2025). This strategy allowed the introduction of new therapeutic modalities, such as aldosterone synthase inhibitors (ASIs) (Calhoun et al. 2011; Gomez-Sanchez and Gomez-Sanchez 2023). One recently introduced medication is lorundrostat, a selective ASI that directly inhibits the production of aldosterone (Pitt and Williams 2024; Kobayashi et al. 2025). Unlike mineralocorticoid receptor antagonists (MRAs) that non-specifically block receptors after aldosterone is produced, often causing off-target effects, lorundrostat targets the enzyme responsible for aldosterone synthesis, leading to more precise blood pressure control with fewer side effects (Pitt and Williams 2024; Kobayashi et al. 2025). However, as the drug was just recently introduced, there is still insufficient data regarding its safety and efficacy, with lacking comprehensive evidence to guide the use of this medication for the treatment of uncontrolled hypertension. Therefore, in this systematic review and meta-analysis aims to synthesize evidence from the published randomized controlled trials (RCTs) to evaluate the efficacy and safety of lorundrostat.

## Methodology

The study was conducted in accordance with the Preferred Reporting Items for Systematic Reviews and Meta-Analyses (PRISMA) statement and Cochrane Handbook for Systematic Reviews and Meta-analysis of Interventions (Cumpston et al. 2019; Page et al. 2021).

A comprehensive search was conducted on PubMed, Scopus, and Web of Science until July 2025 for randomized controlled trials assessing lorundrostat in patients with uncontrolled hypertension.

Since ambulatory blood pressure is a more reliable indicator of blood pressure than office measurement, and office blood pressure is more frequently assessed in clinical practice (Schwartz et al. 2020), our primary outcomes included changes from baseline in both ambulatory and office systolic and diastolic blood pressures.

The pooled categorical variables were represented as a risk ratio (RR) with corresponding 95% confidence intervals (CIs). Continuous variables were represented as mean difference (MD) and the standard deviation (SD). Random effects model (Der Simonian Laird model) was used to analyze the data. Statistical heterogeneity was assessed through the Chi-square test, or Cochran’s *Q* test. A *P* value less than 0.1 suggested the presence of significant heterogeneity with an *I*^2^ value ≥ 50% indicating high heterogeneity. STATA Corp MP 17 was used for analysis. A *P* value of less than 0.05 was considered statistically significant.

## Results

A total of three RCTs, comprising 1568 patients, were included (Laffin et al. 2023, 2025; Saxena et al. 2025). The mean age was 63.5 years, and males comprised 59.1% of the sample population. The mean reported body mass index across the studies was 31.7 kg/m^2^. The follow-up duration of the included studies ranged from 8 to 12 weeks. The full overview of the summary and baseline characteristics of included RCTs are presented in Table 1.

**Table 1:** Characteristics and baseline data of included studies

Study‡	Design	Dose	Patient population	Arm	Number of patients	Age, years	Sex, males	BMI, kg/m^2^	Race				OBP at randomization, mmHg		eGFR at randomization, mL/min/1.73 m^2^	DM	Antihypertensive medications at randomization	
									**White**	**Black or African American**	**Hispanic or Latino**	**Other**	**Systolic**	**Diastolic**			**2**	** ≥ 3**
** Laffin 2023 (The Target-HTN) (Cohort 1) (Laffin et al.**^2023^**)†**	RCT	Lorundrostat 12.5 mg, 50 mg, or 100 mg OD or 12.5 mg or 25 mg BID	Adults aged ≥ 18 years with an automated office SBP of 130 mmHg or greater while taking 2 or more antihypertensive medications for at least 4 weeks at maximally tolerated doses and suppressed plasma (PRA ≤ 1.0 ng/mL/h) and serum aldosterone level of 1.0 ng/dL or greater based on morning measurements	**Lorundrostat 100 mg OD**	30	68.7 (8.9)	12 (40)	30.4 (5.5)	14 (46.7)	15 (50)	16 (53.3)	1 (3.3)	142.2 (13.4)	78.5 (10)	77.4 (14)	8 (26.7)	14 (46.7)	16 (53.3)
				**Lorundrostat 50 mg OD**	28	64.7 (9.5)	13 (46.4)	32 (5)	19 (67.9)	8 (28.6)	14 (50)	0 (0)	140 (12.1)	84.7 (7)	77.2 (14.1)	8 (28.6)	20 (71.4)	8 (28.6)
				**Lorundrostat 25 mg BID**	30	64.8 (9.7)	11 (36.7)	30.6 (5.5)	23 (76.7)	7 (23.3)	17 (56.7)	0 (0)	142.8 (13.1)	80.1 (9.3)	80.9 (12.4)	11 (36.7)	14 (46.7)	16 (53.3)
				**Lorundrostat 12.5 mg BID**	22	68.1 (10.1)	8 (36.4)	32 (5.2)	15 (68.2)	7 (31.8)	7 (31.8)	0 (0)	142.6 (13.3)	81.6 (9.4)	81.7 (16.3)	9 (40.9)	7 (31.8)	15 (68.2)
				**Lorundrostat 12.5 mg OD**	23	65.2 (11.3)	11 (47.8)	30.6 (4.9)	11 (47.8)	11 (47.8)	10 (43.5)	1 (4.3)	142.9 (13.7)	80.3 (12)	77.9 (18.7)	11 (47.8)	14 (60.9)	9 (39.1)
				**Placebo**	30	62.6 (10.7)	13 (43.3)	31.9 (5)	13 (43.3)	16 (53.3)	12 (40)	1 (3.3)	142.9 (10.7)	83.8 (9.5)	81.6 (17.3)	14 (46.7)	17 (56.7)	13 (43.3)
** Laffin 2023 (The Target-HTN) (Cohort 2) (Laffin et al. **2023**)† **		Lorundrostat 100 mg OD	Adults aged ≥ 18 years with an automated office SBP of 130 mmHg or greater while taking 2 or more antihypertensive medications for at least 4 weeks at maximally tolerated doses and PRA of greater than 1.0 ng/mL/h	**Lorundrostat 100 mg OD**	31	66.6 (10.6)	10 (32.3)	30.5 (4.4)	25 (80.6)	6 (19.4)	17 (54.8)	0 (0)	139.8 (9.1)	78.6 (10)	79.9 (13.1)	16 (51.6)	20 (64.5)	11 (35.5)
				**Placebo**	6	62.7 (12.3)	2 (33.3)	32 (3.9)	4 (66.7)	2 (33.3)	2 (33.3)	0 (0)	135.3 (5.5)	81.5 (7.9)	83.9 (18.6)	2 (33.3)	4 (66.7)	2 (33.3)
** Laffin 2025 (The Advance-HTN trial) (Laffin et al. **2025**)† **	RCT	**Dose-adjustment group**: 50 mg of Lorundrostat daily for at least 4 weeks, with an increase to 100 mg daily for the remaining 8 weeks if they met prespecified criteria (office SBP was ≥ 130 mmHg at week 4 and a serum potassium level ≤ 4.8 mmoL/L, a serum sodium level ≥ 135 mmoL/L, an eGFR > 45 mL/min/1.73 m^2^, and a reduction in the eGFR < 25% since randomization) **Stable-dose group**: 50 mg of lorundrostat daily for 12 weeks	Adults aged ≥ 18 years who were receiving stable doses of 2 to 5 antihypertensive medications and had a SBP between 140 and 180 mm Hg and a DBP between 65 and 110 mm Hg, or a DBP between 90 and 110 mmHg regardless of SBP, as measured with an automated device during an office visit	**Lorundrostat 50 mg and then 100 mg OD**	96	60.9 (10.2)	54 (56)	-	35 (36)	56 (58)	9 (9)	5 (5)	143.5 (12.8)	85.6 (10.2)	76.4 (19.4)	46 (48)	59 (61)	37 (39)
				**Lorundrostat 50 mg OD**	94	61.3 (9.6)	56 (60)	-	39 (41)	50 (53)	13 (14)	5 (5)	141.8 (14.4)	84.3 (9.4)	76.6 (17.8)	39 (41)	59 (63)	35 (37)
				**Placebo**	95	59.1 (10.5)	62 (65)	-	41 (43)	44 (46)	15 (16)	10 (11)	141.7 (14)	85.5 (10.3)	73.6 (18)	34 (36)	61 (64)	34 (36)
** Saxena 2025 (The Launch-HTN trial)(Saxena et al. **2025**)† **	RCT	**Group 1**: 50 mg of Lorundrostat OD for 6 weeks if they met prespecified criteria at week 6 (office SBP ≥ 130 mmHg, serum potassium level ≤ 4.8 mmol/L, serum sodium level ≥ 135 mmol/L, eGFR > 45 mL/min/1.73 m^2^, and < 25% reduction in eGFR from baseline) **Group 2**: 50 mg of lorundrostat OD for 12 weeks	Adults aged ≥ 18 years with an unattended automated office SBP of 135 to 180 mm Hg and DBP of 65 to 110 mm Hg or an isolated automated office DBP of 90 to 110 mmHg while taking stable doses of 2 to 5 prescribed antihypertensive medications	**Lorundrostat 50 mg and then 100 mg OD**	270	61.4 (10.3)	142 (52.6)	32.8 (6.7)	190 (70.4)	72 (26.7)	102 (37.8)	5 (1.85)	148.2 (11.9)	87.3 (9.1)	92.8 (17.6)	76 (28.2)	106 (39.3)	164 (60.7)
				**Lorundrostat 50 mg OD**	541	61.7 (10.6)	294 (54.3)	33 (6.9)	366 (67.7)	152 (28.1)	191 (35.3)	15 (2.77)			90.1 (17.8)	173 (32.2)	213 (39.4)	328 (60.6)
				**Placebo**	272	61.8 (10.4)	139 (51.1)	32.6 (7.4)	177 (65.1)	87 (32)	101 (37.1)	7 (2.57)	148.6 (12.1)	87.1 (9.0)	91.2 (16.4)	89 (33)	113 (41.5)	159 (58.5)

†*BID* Twice daily, *BMI* body mass index, *DBP* diastolic blood pressure, *DM* diabetes mellitus, *OBP* office blood pressure, *OD* once daily, *PRA* plasma renin activity, *RCT* randomized controlled trial, and *SBP* systolic blood pressure

‡Dichotomous variables are reported as number and percentage, and continuous variables are reported as mean and standard deviation

**Fig. 1:**
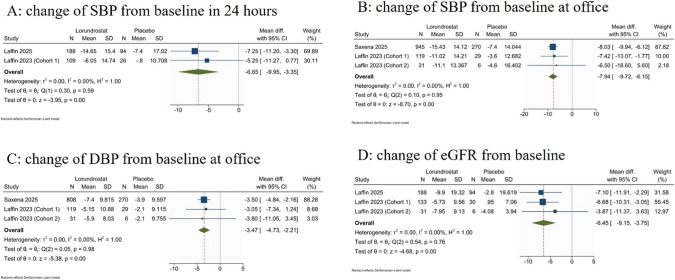
A meta-analysis forest plot using random effects model for **A** the change from baseline in 24-h systolic blood pressure (SBP). **B** Change of SBP from baseline at office, **C** change of diastolic blood pressure (DBP) from baseline at office, **D** change in estimated glomerular filtration rate (eGFR)

Lorundrostat significantly reduced the 24-h systolic blood pressure (SBP) after 8–12 weeks of treatment (MD: − 6.65 mmHg; 95% CI: [− 9.95 to − 3.35], *P* value < 0.001, *I*^2^ = 0%) (Fig. 1A), the change of office SBP from baseline (MD: − 7.94 mmHg; 95% CI: [− 9.72 to − 6.15], *P* value < 0.001, *I*^2^ = 0%) (Fig. 1B), the change of office diastolic blood pressure (DBP) from baseline (MD: − 3.47 mmHg; 95% CI: [− 4.73 to − 2.21], *P* value < 0.001, *I*^2^ = 0%) (Fig. 1C), and the change in estimated glomerular filtration rate (eGFR) (MD: − 6.45 mL/min/1.73m^2^; 95% CI: [− 9.15 to − 3.75], *P* value < 0.001, *I*^2^ = 0%) (Fig. 1D).

**Fig. 2:**
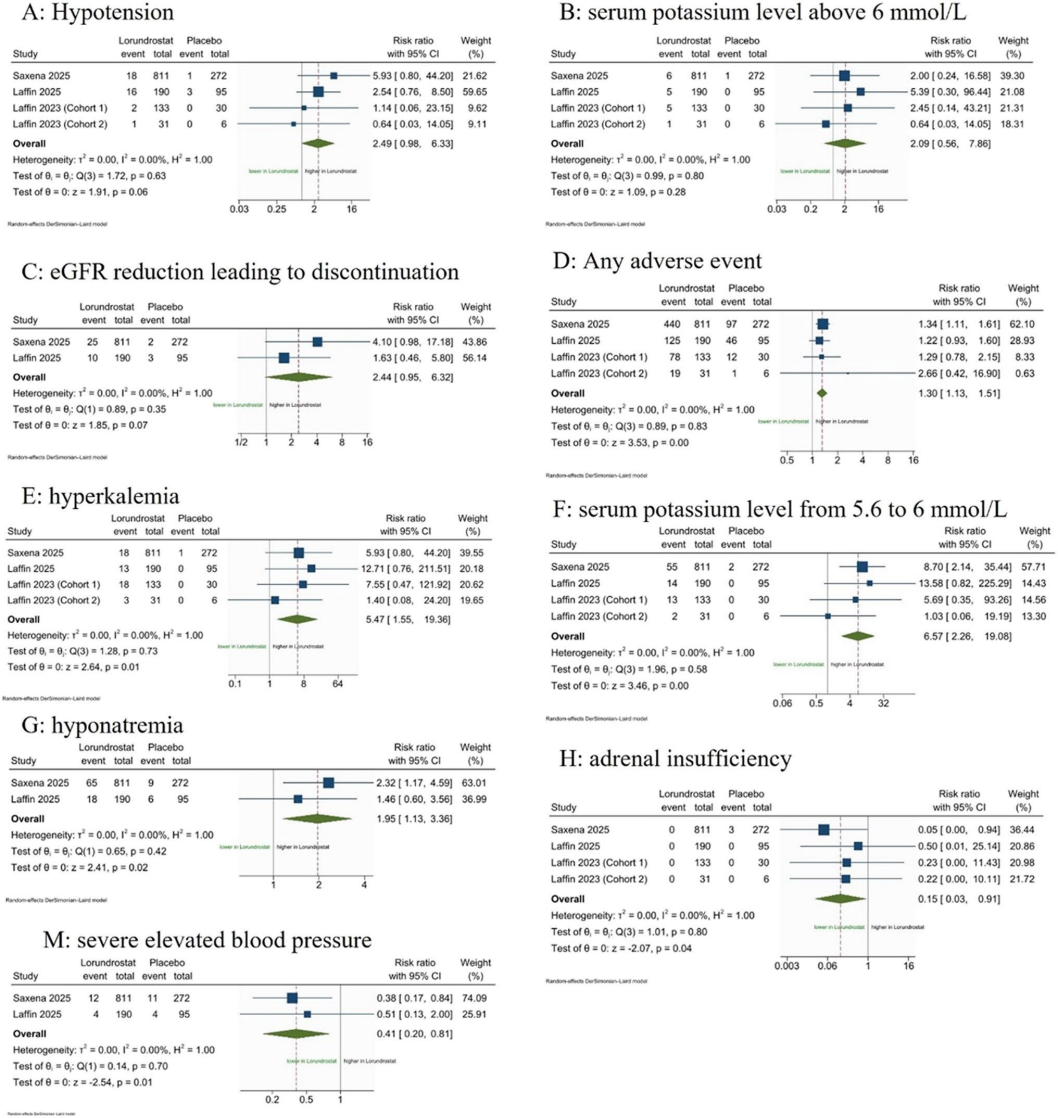
A meta-analysis forest plot using random effects model for safety outcomes: **A** Hypotension, **B** serum potassium level above 6 mmol/L, **C** eGFR reduction leading to discontinuation, **D** any adverse events, **E** hyperkalemia, **F** serum potassium level from 5.6 to 6 mmol/L, **G** hyponatremia, **H** adrenal insufficiency, and **M** severe elevated blood pressure

Regarding the safety outcomes, comparable results between lorundrostat and placebo were shown in hypotension (RR: 2.49; 95% CI: [0.98 to 6.33], *P* value = 0.06, *I*^2^ = 0%) (Fig. 2A), serum potassium level above 6 mmol/L (RR: 2.09; 95% CI: [0.56 to 7.86], *P* value = 0.28, *I*^2^ = 0%) (Fig. 2B), and eGFR reduction leading to discontinuation (RR: 2.44; 95% CI: [0.95 to 6.32], *P* value = 0.07, *I*^2^ = 0%) (Fig. 2C). However, lorundrostat was associated with a higher risk of any adverse events (RR: 1.30; 95% CI: [1.13 to 1.51], *P* value < 0.001, *I*^2^ = 0%) (Fig. 2D), hyperkalemia (RR: 5.47; 95% CI: [1.55 to 19.36], *P* value = 0.01, *I*^2^ = 0%) (Fig. 2E), serum potassium level from 5.6 to 6 mmol/L (RR: 6.57; 95% CI: [2.26 to 19.08], *P* value < 0.001, *I*^2^ = 0%) (Fig. 2F), and hyponatremia (RR: 1.95; 95% CI: [1.13 to 3.36], *P* value = 0.02, *I*^2^ = 0%) (Fig. 2G) compared to placebo at follow-up. Using lorundrostat significantly reduced the risk of adrenal insufficiency (RR: 0.15; 95% CI: [0.03 to 0.91], *P* value = 0.04, *I*^2^ = 0%) (Fig. 2H) and severely elevated blood pressure (RR: 0.41; 95% CI: [0.20 to 0.81], *P* value = 0.01, *I*^2^ = 0%) (Fig. 2M) compared to placebo at follow-up.

## Discussion

To our knowledge, this is the first meta-analysis to assess the efficacy and safety of lorundrostat compared to placebo in patients with resistant hypertension. The analysis demonstrated that lorundrostat significantly reduces both 24-h and office SBP and DBP compared to placebo. This is consistent with the results reported by the three included trials and may be attributed to its mechanism of directly inhibiting aldosterone synthase, thereby reducing excess aldosterone, a key driver of resistant hypertension (Laffin et al. 2023, 2025; Saxena et al. 2025). Regarding renal function, the drug was associated with a small but statistically significant reduction in eGFR. This effect may be explained by decreased aldosterone-mediated sodium retention and changes in renal hemodynamics, which highlights the importance of close surveillance and regular monitoring (Saxena et al. 2025). Regarding safety, lorundrostat showed similar risks of death and hypotension to placebo. Severe hyperkalemia (> 6 mmol/L) and treatment discontinuation due to eGFR decline were not significantly different. However, milder hyperkalemia (5.6–6 mmol/L) and hyponatremia risks were increased, highlighting the need for close electrolyte monitoring during treatment. Notably, the observed fivefold increased risk of hyperkalemia (RR: 5.47) in lorundrostat-treated patients is an important safety concern that warrants careful attention. While severe cases (> 6 mmol/L) were not significantly more frequent, the absolute increase in moderate hyperkalemia emphasizes the necessity for strict monitoring of serum potassium, particularly in populations at higher baseline risk such as those with chronic kidney disease, diabetes, or concomitant RAAS inhibitors. The significant reduction in eGFR, though numerically modest, may portend risk for patients with marginal renal reserve, suggesting that lorundrostat should be used with caution in these groups and that safety surveillance protocols must be clearly established in clinical practice. These findings underscore that, while the drug is efficacious for lowering blood pressure, its deployment should always be coupled with routine renal and electrolyte assessment, especially during initiation and dose escalation. These findings align with the pharmacological profile of aldosterone antagonists that can cause electrolyte imbalances due to their effects on renal sodium and potassium handling (Nappi and Sieg 2011). Across included studies (Laffin et al. 2023, 2025; Saxena et al. 2025), no cases of adrenal insufficiency were reported in the lorundrostat groups, while a small number occurred in placebo (pooled RR 0.15, 95% CI 0.03–0.91). This may be attributed to lorundrostat’s selective inhibition of aldosterone synthase (CYP11B2) while sparing 11β-hydroxylase (CYP11B1), thus minimizing cortisol suppression. As a result, the apparent reduction in adrenal insufficiency may reflect true endocrine selectivity; however, these findings are based on very few events and should be interpreted with caution pending confirmatory studies (Freeman et al. 2023; Irfan et al. 2024). These findings substantiate the recommendations in the recent consensus statements in ESC and JSH, which advocate for stringent blood pressure control at levels below 130/80 mmHg to mitigate cardiovascular risk (Zuin et al. 2025; Ohya and Sakima 2025). The statements specifically advocate for the early diagnosis of primary aldosteronism and individualized therapy through selective aldosterone synthase inhibition, with lorundrostat being positioned as the leading therapeutic agent. Routine assessment of primary aldosteronism is also recommended, thereby augmenting lorundrostat’s established therapeutic benefit in the population with resistant hypertension (Burlacu et al. 2025). The efficacy of lorundrostat is supported by extensive trial data. Significant SBP drops in over 1000 treatment-resistant hypertensives were reported in phase 3 launch-HTN, with good tolerability and adverse events at low levels (Saxena et al. 2025). Phase 2 advance-HTN demonstrated consistent 24-h SBP reductions at 12 weeks in difficult-to-manage populations (Laffin et al. 2025). Further, the TARGET-HTN titration study presented dose-related systolic reductions, averaging over 14 mmHg at clinic visits, and noted primarily mild and short-term increases in serum potassium as the main adverse effect (Laffin et al. 2023).

Our data, consistent with those by Siddiqui et al. recorded mean reductions near 6.5 to 8-mmHg SBP and 3.5-mmHg DBP, with low heterogeneity (Siddiqui et al. 2024). A recent meta-analysis by Goh et al., which included eight randomized controlled trials and over 2600 patients, provides a broad overview of aldosterone synthase inhibitor efficacy in resistant hypertension (Goh et al. 2025). Their pooled data echoed our findings, reporting consistent reductions in systolic and diastolic blood pressure and similar trends toward increased risk of hyperkalemia and modest reductions in eGFR. However, Goh et al. incorporated a more diverse set of study populations and longer follow-up periods, which allowed for additional subgroup analyses relating to baseline renal function and ethnicity. In contrast, our meta-analysis offers a focused evaluation of lorundrostat specifically, using detailed safety endpoints and standardized definitions of adverse events. These values fall within the anticipated class effect and replicate previously reported blood pressure and safety profiles, the latter of which being dominated by cases of mild hyperkalemia. Some earlier studies have indeed highlighted electrolyte disturbances with ASIs, particularly hyperkalemia and an acute decline in eGFR (Awosika et al. 2023). We confirmed an elevated relative risk in both parameters, but the incidence of severe hyperkalemia and the rate of treatment cessation due to renal impairment were not substantially higher in the lorundrostat group. However, it is important to recognize that several adverse events were more frequent in the lorundrostat group but did not reach statistical significance, likely due to limited power from including only three or fewer trials. This limitation should be acknowledged and highlights the need for further research with larger sample sizes to definitively assess these safety outcomes. Variability in safety profiles across individual analyses may arise from differences in study samples, dose regimens, duration of follow-up, and the categories employed to define adverse events. The latest RCTs may have inadvertently underestimated the risk to the cardiovascular system because the recruited patients were heterogeneous, and follow-up was brief (mean of 10 weeks). The more pronounced and sustained decrease in adrenal insufficiency and severe hypertensive complications observed in our analysis, though reported inconsistently in previous meta-analyses (Shimizu et al. 2024), may be attributable to lorundrostat’s selective inhibition of aldosterone synthase (CYP11B2) without significant cortisol synthesis inhibition, as demonstrated in first-in-human pharmacokinetic and safety data (Freeman et al. 2023; Irfan et al. 2024). Such selectivity may provide endocrine benefits absent in less selective ASIs or MRAs and may underline the protective effect against adrenal insufficiency as a key clinical advantage.

## Strength and limitations

This meta-analysis combines data from three well-designed clinical trials, all featuring considerable sample sizes, which bolsters the accuracy and generalizability of the lorundrostat’s efficacy and safety profile in uncontrolled hypertension. The use of common outcome measures along with low heterogeneity also improves the certainty of the results. In addition, the small number of included studies is insufficient for robust subgroup analyses and comprehensive safety evaluations. Additionally, the mean follow-up period of pooled studies was short, averaging 10 weeks, which is not sufficient to assess the persistence of efficacy on the long-term, and it may underestimate the long-term renal risk and trajectory of hyperkalemia; larger and longer trials are needed to further clarify the drug’s long-term safety and efficacy profiles.

## Recommendation of future research

Future research should intensify safety surveillance, with particular focus on surveillance of renal function, electrolyte homeostasis, and the anticipated antihypertensive effect of lorundrostat, extending the duration of follow-up period. To enhance generalizability, studies should intentionally include representative ethnic groups and a full spectrum of baseline renal function in real-world circumstances. Simultaneous analyses comparing the effectiveness of lorundrostat against existing aldosterone inhibitors and baseline antihypertensives will assist in defining its role in the therapeutic spectrum. Supporting mechanistic studies is warranted to clarify the precise adrenal modulation and the resultant cardiovascular implications extending beyond the measurable change in systemic arterial pressure. For cohorts presenting resistant and uncontrolled hypertension, upcoming evaluations ought to bundle markers of adherence to therapy, patient-reported outcomes, and quality-of-life data, thereby embedding lorundrostat in a comprehensive assessment of value in contemporary treatment platforms.

## Conclusion

Lorundrostat may be an effective agent for lowering blood pressure in patients with uncontrolled hypertension. Its safety profile is acceptable but requires careful monitoring of serum electrolytes and renal function. The reduced risk of adrenal insufficiency highlights its selectivity and potential as a valuable therapeutic option. Further large-scale, long-term studies are needed to confirm its sustained benefits and safety.

## Data Availability

Not applicable.
